# Pediatric RVOT reconstruction with ePTFE trileaflet valved conduits: a dual-center Chinese study

**DOI:** 10.3389/fcvm.2024.1447487

**Published:** 2024-09-18

**Authors:** Kai Luo, Qi-Liang Zhang, Xiao-Yang Zhang, Zi-Jie Zhou, Yan-Jun Pan, Zhong-Qun Zhu, Qiang Chen, Jing-Hao Zheng, Xiao-Min He, Wei Zhang

**Affiliations:** ^1^ Department of Cardiothoracic Surgery, Shanghai Children’s Medical Center, Heart Center, School of Medicine, Shanghai Jiaotong University, Shanghai, China; ^2^ Department of Cardiac Surgery, Fujian Children’s Hospital (Fujian Branch of Shanghai Children’s Medical Center), College of Clinical Medicine for Obstetrics & Gynecology and Pediatrics, Fujian Medical University, Fuzhou, China

**Keywords:** polytetrafluoroethylenep, trileaflet valved conduits, pediatric, right ventricular outflow tract reconstruction, pulmonary valve regurgitation

## Abstract

**Objective:**

This study aims to assess the early to mid-term clinical efficacy of expanded polytetrafluoroethylene (ePTFE) trileaflet valved conduits in pediatric right ventricular outflow tract reconstruction for congenital heart disease.

**Methods:**

We conducted a retrospective analysis of pediatric patients who underwent right ventricular outflow tract (RVOT) reconstruction using ePTFE trileaflet valved conduits at two cardiac centers in China, between January 2017 and June 2023. The main assessment criterion was the functionality of the prosthetic pulmonary valve conduit.

**Results:**

A total of 162 pediatric patients were included, with follow-up periods ranging from 0.1 to 5 years post-discharge, and a median follow-up duration of 1 year (interquartile range: 1, 2). Three patients (1.9%) required re-operation due to conduit obstruction. During follow-up, pulmonary valve flow velocities were recorded as <3 m/s in 134 patients (82.7%), between 3 and 4 m/s in 24 patients (14.8%), and >4 m/s in 4 patient (2.5%). Mild pulmonary valve regurgitation was noted in 148 patients (91.4%), and moderate pulmonary valve regurgitation was noted in 14 patients (8.6%), with no instances of more than moderate pulmonary valve regurgitation.

**Conclusion:**

The ePTFE trileaflet valved conduit, known for its accessibility and simplicity in manufacturing, demonstrates favorable early to mid-term clinical outcomes in pediatric RVOT reconstruction.

## Introduction

Right ventricular outflow tract (RVOT) reconstruction is a critical surgical intervention aimed at mitigating right ventricular outflow tract obstructions and enhancing pulmonary valve function (1, 2). This procedure is essential in treating various congenital heart conditions such as Tetralogy of Fallot (TOF), TOF with double outlet right ventricle, pulmonary stenosis, pulmonary atresia, and persistent truncus arteriosus, as well as in cases requiring the ROSS procedure or reoperations due to progressive pulmonary valve regurgitation or RVOT obstruction. An optimal RVOT reconstruction should effectively alleviate the obstruction while preserving or improving pulmonary valve function over time. However, the inherent complexities of these cardiac conditions and the limitations of existing reconstruction materials often result in less-than-ideal outcomes (3–6). Although valved conduits are a standard material in these surgical procedures, continuous innovations in surgical techniques and material development have yet to significantly improve long-term outcomes (3–6).

Expanded polytetrafluoroethylene (ePTFE) is a material with numerous advantages for surgical applications, including a smooth surface, low friction coefficient, durability, excellent tissue compatibility, minimal biological reaction, and resistance to deformation, degradation, calcification, and inflammation during prolonged implantation (7). In the past decade, hand-sewn ePTFE valved conduits have been increasingly utilized in RVOT reconstruction surgeries. These conduits have demonstrated satisfactory clinical outcomes and acceptable graft failure rates (8–14). However, literature regarding the use of ePTFE valved conduits within mainland China remains limited. This study aims to consolidate the experiences of two cardiac centers in mainland China in employing ePTFE trileaflet valved conduits for pediatric RVOT reconstruction, highlighting the early to mid-term clinical results.

## Methods

This retrospective study analyzed clinical data from 169 pediatric patients who underwent ROVT using ePTFE trileaflet valved conduits at two cardiac centers between January 2017 and June 2023. Eligibility for inclusion required patients to have undergone RVOT reconstruction using a ePTFE trileaflet valved conduit. Exclusion criteria were postoperative mortality, incomplete clinical data, or the patient's family declining participation in the study. Ethical approval for this study was granted by the respective hospital review boards, and informed consent was obtained from the families of all participating patients.

The assessment of conduit stenosis was conducted using continuous-wave Doppler, measuring the maximum flow velocity through the conduit and the resulting pressure gradient across the right ventricular outflow tract, in accordance with the Bernoulli equation. The stenosis was categorized into three levels: mild (peak velocity up to 3 m/s or peak gradient up to 36 mmHg), moderate (peak velocity between 3 and 4 m/s, peak gradient between 36 and 64 mmHg), and severe (peak velocity exceeding 4 m/s or peak gradient surpassing 64 mmHg). Pulmonary valve regurgitation was classified into three grades: trivial to mild (encompassing trivial, trivial to mild, and mild), mild to moderate (reflux image confined within the conduit), and moderate to severe (categories include moderate, moderate to severe, severe; with reflux image extending into the sinus of the right ventricle). The grading criteria employed were aligned with established guidelines in echocardiography (15).

### ePTFE trileaflet valved conduits fabrication method

**Figure 1:**
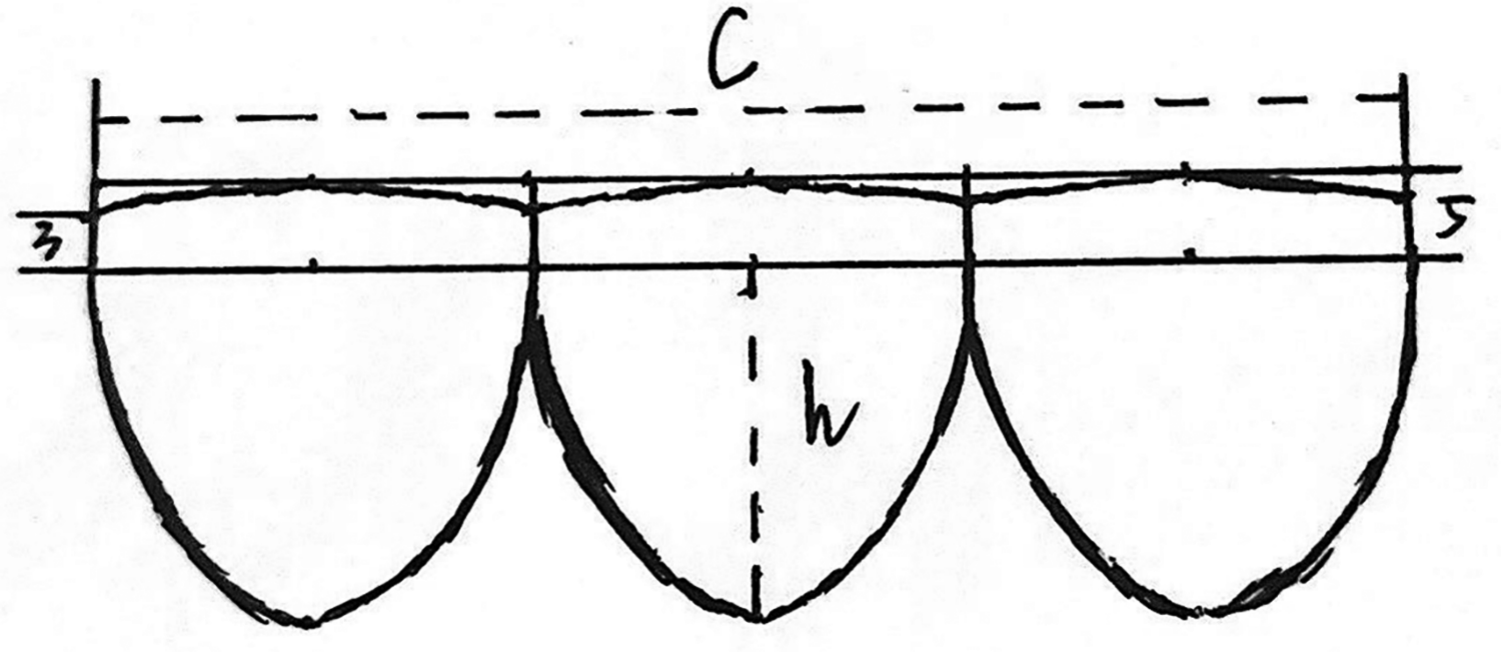
This figure presents a schematic diagram illustrating the design of the valve leaflets. The free edge of each leaflet is aligned in a straight line, while the bottom edge of the valvular sinus forms an appropriate arc. Each leaflet is of equal length, with their combined length equaling the conduit's circumference (c=π × d). The leaflet height is set at 4/5 of the conduit's diameter (h = 0.8 × d). The bottom margin of the valvular sinus is a symmetrical arc, with the central and free margin heights being 3 mm and 5 mm, respectively.

**Figure 2:**
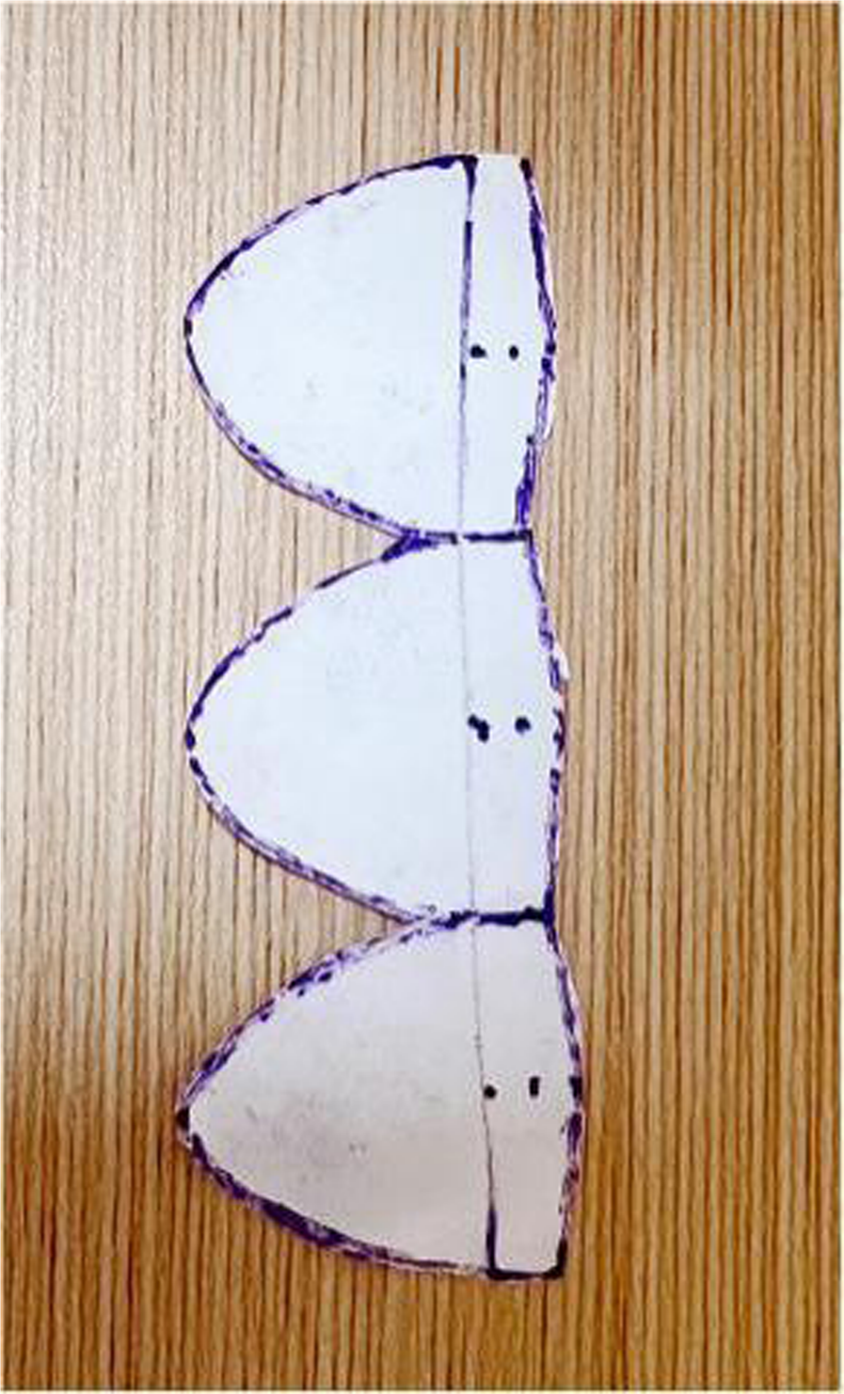
This figure shows the process of cutting out the valve leaflets as per the schematic diagram.

The construction of the valved conduit was carried out intraoperatively. Initially, the design of the valve leaflets was traced onto an ePTFE pericardial patch provided by W.L. Gore & Associates Inc (Figure 1). The total length of the leaflets’ free edge was made equal to the circumference of the selected Gore-Tex graft, and it was divided into three equal segments to create three leaflets, each measuring one-third of the circumference (1/3C). The height of the valve sinus was determined to be four-fifths of the conduit's diameter. The free edges of the leaflets were aligned at the same level. The bottom edge of the valve sinus was designed as a symmetrical arc, with the central heights at the base and free edge being 3 mm and 5 mm, respectively (Figure 2).

**Figure 3:**
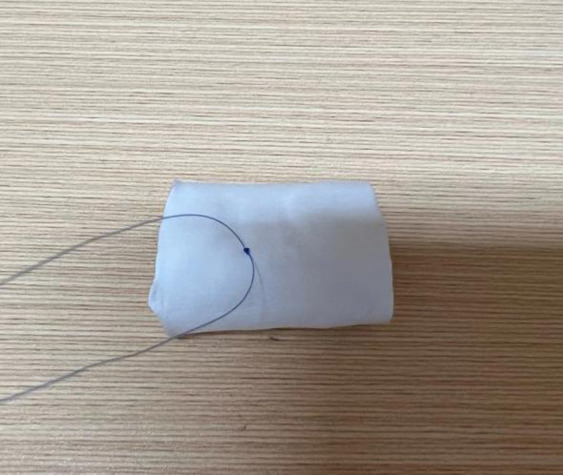
The image depicts the starting of the needlework from the junction where the two ends of the valve leaflets meet. A knot is tied at this starting point to secure the suture.

**Figure 4:**
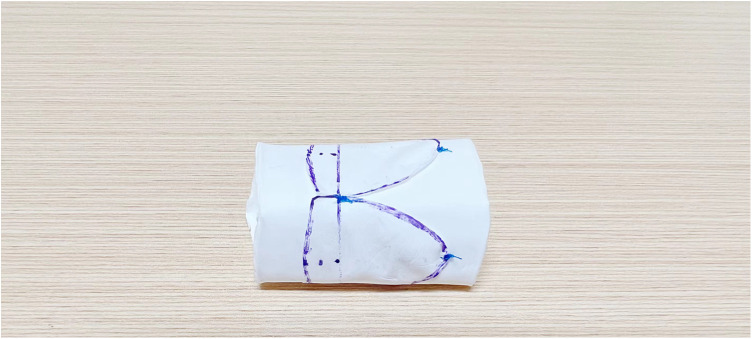
This figure illustrates the continuous and even suturing of the valve leaflets to the inner surface of the vessel. The junction of the valve leaflet is reinforced with round-trip suturing for added strength.

**Figure 5:**
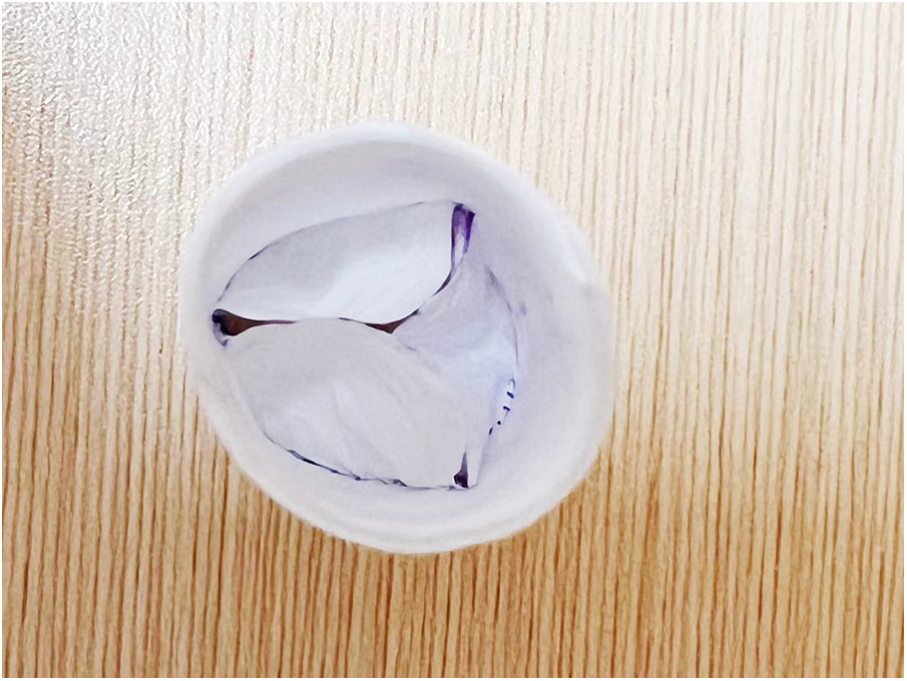
The figure displays the conduit being turned over to inspect the shape and placement of the valve leaflets post-suturing.

The maximal conduit size of the Gore-Tex graft (from W.L. Gore & Associates Inc) was calculated using standard z-score tables by patient’s weight and height. To prepare for suturing, the graft was turned inside out to accurately mark the suture site, ensuring that the free edges of the valve leaflets were aligned at the same level to prevent distortion. The leaflets were then sutured with 5-0 Prolene, beginning at the junction of both ends and tying a knot at this starting point upon completion (Figure 3). Suturing of the leaflets to the graft's inner surface was performed continuously and evenly, maintaining a stitch spacing of approximately 1.0 mm, and careful to avoid penetrating the graft wall. The junction points of the leaflets received additional reinforcement through back-and-forth suturing (Figure 4). Post-sewing, the valved conduit was inverted again to examine the leaflets’ formation (Figure 5) (16).

### Right ventricular outflow tract reconstruction method

Following anesthesia induction, a median sternotomy was performed to establish cardiopulmonary bypass. Cardiac arrest was induced by clamping the aorta, allowing for the correction of intracardiac anomalies. Once the aortic clamp was removed and the heart began beating again, the RVOT reconstruction was carried out while the heart continued to beat under parallel circulation. In cases where significant stenosis was present in the left and right pulmonary arteries, a pericardial patch was utilized for enlargement. For significant aneurysmal dilation, appropriate folding techniques were employed to achieve the desired shape. During the RVOT reconstruction, the distal end of the valved conduit was anastomosed end-to-end to the pulmonary artery confluence using 5-0 or 6-0 Prolene sutures. Care was taken to minimize the length of the distal end of the conduit (usually preserving 5–10 mm above the valve), ensuring that the distal anastomosis remained clear of the pulmonary artery confluence and preventing twisting or angulation of the conduit. Subsequently, the proximal end of the conduit was obliquely trimmed and anastomosed to the right ventricular infundibulum.

### Data collection

Data was collected from inpatient medical records, encompassing a range of categories. Preoperative data included patient demographics (age, sex, weight), diagnosis, and any pre-existing comorbidities. Perioperative data covered key surgical details such as cardiopulmonary bypass duration, type of conduit used, length of stay in the intensive care unit, overall hospitalization duration, and any postoperative complications. Follow-up data included the duration of follow-up and postoperative assessments of pulmonary valve stenosis, pulmonary valve regurgitation, and tricuspid valve regurgitation.

### Management of follow-up

Postoperatively, patients routinely receive anticoagulation therapy for one year. The therapeutic regimen includes either warfarin, aiming to maintain an International Normalized Ratio (INR) between 1.5 and 2.0, or aspirin at a dosage of 5 mg/(kg·d). Echocardiography and electrocardiogram were reviewed at 1 month, 3 months, 6 months, and 1 year after discharge, and then annually.

### Statistical analyses

Data analyses were performed using IBM SPSS Statistics version 25.0 (SPSS, Inc., Chicago, IL.). Descriptive continuous variables were presented as median with interquartile range (IQR); categorical variables were presented as numbers or percentage.

## Results

In this study, 168 pediatric patients were initially included. However, 6 were lost to follow-up post-discharge, resulting in the analysis of 162 patient records. The breakdown of conditions treated was as follows: 68 initial surgeries, comprising 18 cases of pulmonary atresia, 5 of complete transposition of the great arteries (TGA), 1 of pulmonary insufficiency, 11 of corrected TGA, 2 of persistent truncus arteriosus, 6 of double outlet right ventricle (DORV), and 25 undergoing the ROSS procedure for aortic valve disease. The remaining 94 cases were reoperations, involving patients with pulmonary valve regurgitation or residual RVOT obstruction following previous cardiac surgeries. These included 35 post-pulmonary atresia surgeries, 7 post-pulmonary stenosis surgeries, 43 post-TOF surgeries, 6 post-persistent truncus arteriosus surgeries, 2 post-DORV surgeries, and 1 post-complete TGA surgery. The cohort comprised 93 males and 69 females, with a median age of 8 years (IQR: 4.7, 12), weight 23.5 kg (IQR: 14.9, 39.3), conduit size 21 mm (IQR: 19, 22), cardiopulmonary bypass time 142 min (IQR: 102, 189), intensive care unit stay of 4 days (IQR: 3, 6), and hospital stay of 20 days (IQR: 15, 27). Early postoperative complications were observed as follows: low cardiac output syndrome in 20 patients (12.3%), arrhythmias in 4 patients (2.5%), infective endocarditis in 2 patients (1.2%), gastrointestinal bleeding in 2 patients (1.2%), renal failure in 3 patients (1.9%), elevation of the diaphragm in 1 patient (0.6%), and chylothorax in 1 patient (0.6%) (Table 1).

**Table 1:** Medical data of the patients.

Item	
Number	162
Age (year)	8 (4.7, 12)
Weight (kg)	23.5 (14.9, 39.3)
Boys/Girls	93/69
Size of conduit (mm)	21 (19, 22)
Reoperation of right ventricular outflow tract reconstruction	94 (58.02%)
Cardiopulmonary bypass time (min)	142 (102, 189)
Intensive care time (day)	4 (3, 6)
length of hospital stay (day)	20 (15, 27)
Postoperative complications	
Low cardiac output syndrome	20 (12.3%)
Arrhythmia	4 (2.5%)
Infective endocarditis	2 (1.2%)
Hemorrhage of digestive tract	2 (1.2%)
Renal failure	3 (1.9%)
Diaphragmatic eventeration	1 (0.6%)
Chylothorax	1 (0.6%)
Follow-up time (year)	1 (1, 2)
Moderate tricuspid valve regurgitation	4 (2.5%)
More than moderate tricuspid valve regurgitation	0

The follow-up duration ranged from 0.1 to 5 years, with a median follow-up time of 1 year (IQR: 1, 2). For patients with follow-up time ≤ 1 year, pulmonary valve flow rate of 90.2% patients less than 3 m/s, pulmonary valve flow rate of 8.6% patients was between 3 and 4 m/s, and pulmonary valve flow rate of 1.2% patients was more than 4 m/s. 90.2% patients were mild pulmonary valve regurgitation, 9.8% patients were moderate pulmonary valve regurgitation, and no patient was more than moderate pulmonary valve regurgitation. For patients with 1year < Follow-up time ≤ 2 years, pulmonary valve flow rate of 85.4% patients less than 3 m/s, pulmonary valve flow rate of 12.2% patients was between 3 and 4 m/s, and pulmonary valve flow rate of 2.4% patients was more than 4 m/s. 92.6% patients were mild pulmonary valve regurgitation, 7.4% patients were moderate pulmonary valve regurgitation, and no patient was more than moderate pulmonary valve regurgitation. For patients with 2years < Follow-up time ≤3 years, pulmonary valve flow rate of 85% patients less than 3 m/s, pulmonary valve flow rate of 15% patients was between 3 and 4 m/s, and no patient was pulmonary valve flow rate more than 4 m/s. 95% patients were mild pulmonary valve regurgitation, 5% patients were moderate pulmonary valve regurgitation, and no patient was more than moderate pulmonary valve regurgitation. For patients with 3years < Follow-up time ≤4 years, pulmonary valve flow rate of 100% patients less than 3 m/s, and no patient was pulmonary valve flow rate more than 3 m/s. 83.3% patients were mild pulmonary valve regurgitation, 16.7% patients were moderate pulmonary valve regurgitation, and no patient was more than moderate pulmonary valve regurgitation. For patients with 4years < Follow-up time ≤5 years, pulmonary valve flow rate of 15.4% patients less than 3 m/s, pulmonary valve flow rate of 69.2% patients was between 3 and 4 m/s, and pulmonary valve flow rate of 15.4% patients was more than 4 m/s. 92.3% patients were mild pulmonary valve regurgitation, 7.7% patients were moderate pulmonary valve regurgitation, and no patient was more than moderate pulmonary valve regurgitation. During the follow-up period, only 3 patients (1.9%) required re-operation. Two patients developed thrombosis due to regular use of anticoagulants after operation, which resulted in obstruction. One patient required re-operation after 5 years due to narrowing of the conduit caused by insufficient growth of the conduit (Table 2).

**Table 2:** Medical data of the patients in the follow-up time.

	Follow-up time ≤ 1 year	1 year < Follow-up time ≤ 2 years	2 years < Follow-up time ≤3 years	3 years < Follow-up time ≤4 years	4 years < Follow-up time ≤5 years	Total
Number	82	41	20	6	13	162
Pulmonary valve flow rate <3 m/s	74 (90.2%)	35 (85.4%)	17 (85%)	6 (100%)	2 (15.4%)	134 (82.7%)
Pulmonary valve flow rate 3–4 m/s	7 (8.6%)	5 (12.2%)	3 (15%)	0	9 (69.2%)	24 (14.8%)
Pulmonary valve flow rate >4 m/s	1 (1.2%)	1 (2.4%)	0	0	2 (15.4%)	4 (2.5%)
Mild pulmonary valve regurgitation	74 (90.2%)	38 (92.6%)	19 (95%)	5 (83.3%)	12 (92.3%)	148 (91.4%)
Moderate pulmonary valve regurgitation	8 (9.8%)	3 (7.4%)	1 (5%)	1 (16.7%)	1 (7.7%)	14 (8.6%)
More than moderate pulmonary valve regurgitation	0	0	0	0	0	0
Re-operation	1 (1.2%)	1 (2.4%)	0	0	1 (7.6%)	3 (1.9%)

## Discussion

RVOT reconstruction is a vital element in various complex congenital cardiac surgeries. Traditionally, the focus was primarily on comprehensive de-obstruction. However, as clinical experience accumulates, surgeons are increasingly recognizing that thorough de-obstruction of the RVOT, without concurrent pulmonary valve protection, can precipitate significant pulmonary valve regurgitation, adversely affecting right ventricular function. Increased right ventricular volume load can lead to diastolic dysfunction and myocardial fibrosis, resulting in progressive dilation and diverse signs of right ventricular failure. In severe cases, this can lead to major arrhythmias and even sudden cardiac death (17–20). Simultaneous reconstruction of the pulmonary valve function during these procedures can markedly mitigate these complications and preserve right ventricular function. Presently, there is a growing consensus among experts towards incorporating pulmonary valve intervention in RVOT reconstruction (21).

The choice of materials for pulmonary valve prostheses, particularly in pediatric and adolescent patients, remains a topic of ongoing debate. Long-term concerns, including valve calcification and the subsequent need for reoperation, are significant issues. Several materials are available for pulmonary valve prostheses, such as bioprosthetic valves, homografts, mechanical valves, and hand-sewn polytetrafluoroethylene valves. Bioprosthetic valves, which are the most commonly used, are widely accessible and do not necessitate permanent anticoagulation therapy. Their major drawback, however, is a limited lifespan due to structural degradation, especially leaflet calcification, which ultimately leads to valve failure and the need for replacement (22, 23). Mechanical valves, while more durable than bioprosthetic and homograft valves, present the inherent risk of serious bleeding events associated with long-term anticoagulation, a factor that can negatively impact the quality of life in children and young adults (24, 25). The full implementation of artificial bioprosthetic and mechanical valves in pediatric patients is currently hindered by commercialization factors. Homografts, although used, have limited availability and, similar to bioprosthetic valves, are susceptible to long-term failure (26).

ePTFE sheets are known for their outstanding elasticity and flexibility. The unique structural properties of ePTFE inhibit cell adhesion and growth, effectively preventing the deposition of fibrous tissue. This makes ePTFE an ideal material for valve construction. The utilization of ePTFE grafts and sheets in crafting hand-sewn valved conduits presents a promising solution to the challenges associated with homografts, which include limited availability, high costs, a restricted range of sizes, and less than optimal long-term performance *in vivo*. A significant advantage of ePTFE-based materials is that they do not necessitate long-term anticoagulation therapy.

A multicenter study in Japan utilizing ePTFE trileaflet valved conduits for pediatric RVOT reconstruction reported exceptional outcomes (9). The research team led by Yamagishi and Miyazaki conducted extensive studies on ePTFE in RVOT reconstruction, revealing a 15-year freedom from replacement rate of 84.2% (27). A separate analysis focusing on ePTFE valved conduits with diameters over 16 mm showed freedom from replacement rates of 99.5%, 89.0%, and 86.1% at 5, 10, and 15 years, respectively (28). However, there is a relative paucity of data from regions outside Japan. Te-I Chang et al. reported findings from 55 cases using ePTFE trileaflet valved conduits in pediatric RVOT reconstruction, noting a median follow-up of 2.9 years (up to 10 years) with a 10-year freedom from reintervention rate of 98.0%. Postoperative echocardiography indicated mild or trivial pulmonary artery stenosis and pulmonary valve regurgitation in 92.2% and 92.0% of patients, respectively (16). Shi and colleagues, in their report on 72 cases with a median follow-up of 33 months, also observed favorable outcomes, with only 5 cases of conduit dysfunction, including 4 with moderate stenosis and 1 with moderate regurgitation (29). Similar to these findings, the results of our study also showed satisfactory hemodynamic performance and early to mid-term clinical results of ePTFE trileaflet valved conduits in children underwent RVOT reconstruction.

The ePTFE trileaflet valved conduit offers several key benefits. Its symmetric trileaflet design closely mimics the natural structure of the pulmonary semilunar valve, which significantly reduces the risk of pulmonary valve regurgitation. The construction technique, involving the inversion and internal sewing of the leaflets, is straightforward and maintains the structural integrity of the conduit. Additionally, the use of a continuous single-line suture simplifies the assembly process, enhancing the potential for broader application. The design incorporates a 5 mm arcuate valsalva sinus at the base, augmenting its anti-regurgitation efficacy. This arcuate configuration of the conduit is in alignment with the physiological structure of the main pulmonary artery, thereby effectively preventing the formation of angles that could increase blood flow resistance. While higher sinus heights can improve anti-regurgitation properties, the limited space available in pediatric patients and the necessity for the conduit to adopt an arcuate shape mean that conduits with excessively high sinuses might not be feasible for implantation. Based on our experience, a sinus height of 0.8 times the conduit's diameter is typically chosen. Although the ePTFE trileaflet valved conduit works well, pulmonary regurgitation is eventually inevitable. The reasons for postoperative pulmonary regurgitation are as follows. First, the conduit is manually sutured, and there are individual differences. If the suture is not strictly in accordance with the standard, the anti-reflux effect of the conduit would be affected. If the suture valve occurs avulsion, the reflux will also occur necessarily. Eventually time migration, calcification and contracture are also factors that lead to the inevitability of reflux. At the same time, because the artificial conduit can not grow, as the child grows, the problem of conduit stenosis is inevitable, especially for younger child. Therefore, the appropriate maximum diameter of the conduit should be selected as far as possible, and the suture conduit must be strictly in accordance with the standard. It is still necessary to review regularly after operation to pay attention to the functional changes of the conduit.

While the ePTFE trileaflet valved conduit is noted for its excellent tissue compatibility and does not necessitate lifelong anticoagulation like mechanical valves, standard anticoagulation therapy post-implantation is essential to prevent thrombotic events that could result in conduit obstruction. There is no unified standard for postoperative anticoagulation, and the method and time of anticoagulation reported by various studies are different. The method of anticoagulation of Te-I Chang et al. was using Warfarin accordingly to weight to keep INR around 1.5 in the first three months postoperatively. Then anticoagulants were tapered to 1 mg per day with addition of Aspirin 100 mg per day in most adolescent and adult patients (16). The method of anticoagulation of Huifeng Zhang et al. was that warfarin was started as a replacement for heparin, so that the INR reached 1.5–2.0 during the subsequent three months. Oral aspirin was then prescribed for 2 years (30). The method of anticoagulation of Kwang Ho Choi et al. was that all patients received heparin immediately after cessation of postoperative bleeding. Anticoagulation therapy with warfarin was then started after oral intake resumed and was continued for 3 months. Aspirin was administered thereafter (7). The method of anticoagulation of Masahiro Koh et al. was that all were given warfarin sodium and low-dose antiplatelet drugs for at least 1 year after conduit implantation (31). Because of the multiple treatment groups in our study, the anticoagulation regimen was not identical for each treatment group. Patients routinely receive anticoagulation therapy for one year after operation. The therapeutic regimen included either warfarin, aiming to maintain the INR between 1.5 and 2.0, or aspirin at a dosage of 5 mg/(kg·d). Our study showed that the results of anticoagulation were still good, only 2 patients developed thrombosis. The reason of thrombosis was poor compliance and irregular use of anticoagulant drugs.

This study presents several limitations. 1. While it encompasses a relatively large sample size from two centers, the derivation of more objective clinical outcomes would benefit from multicenter studies from multiple countries. 2. The follow-up duration in this study is relatively short. As a result, the long-term clinical efficacy of the intervention requires validation through extended follow-up studies. 3. Given that the ePTFE trileaflet valved conduits are hand-sewn, there is an inherent variability in their functionality, which can be attributed to differences in the craftsmanship of individual makers.

## Conclusion

The application of ePTFE trileaflet valved conduits in pediatric RVOT reconstruction has proven beneficial, yielding favorable clinical outcomes in the early to mid-term stages. The availability and ease of construction of these materials render them a highly effective option for such surgical procedures.

## Data Availability

The original contributions presented in the study are included in the article/Supplementary Material, further inquiries can be directed to the corresponding authors.
