# Corrigendum: Pediatric RVOT reconstruction with ePTFE trileaflet valved conduits: a dual-center Chinese study

**DOI:** 10.3389/fcvm.2024.1499335

**Published:** 2024-10-18

**Authors:** Kai Luo, Qi-Liang Zhang, Xiao-Yang Zhang, Zi-Jie Zhou, Yan-Jun Pan, Zhong-Qun Zhu, Qiang Chen, Jing-Hao Zheng, Xiao-Min He, Wei Zhang

**Affiliations:** ^1^ Department of Cardiothoracic Surgery, Shanghai Children's Medical Center, Heart Center, School of Medicine, Shanghai Jiaotong University, Shanghai, China; ^2^ Department of Cardiac Surgery, Fujian Children's Hospital (Fujian Branch of Shanghai Children's Medical Center), College of Clinical Medicine for Obstetrics & Gynecology and Pediatrics, Fujian Medical University, Fuzhou, China

**Keywords:** polytetrafluoroethylenep, trileaflet valved conduits, pediatric, right ventricular outflow tract reconstruction, pulmonary valve regurgitation

A Corrigendum on Pediatric RVOT reconstruction with ePTFE trileaflet valved conduits: a dual-center Chinese study By Luo K, Zhang Q-L, Zhang X-Y, Zhou Z-J, Pan Y-J, Zhu Z-Q, Chen Q, Zheng J-H, He X-M and Zhang W (2024). Front. Cardiovasc. Med. 11:1447487. doi: 10.3389/fcvm.2024.1447487

In the published article, there was an error in Figure 2 and Figure 3 as published. The figures were the same. The corrected Figure 2 and Figure 3 as well as the captions appear below.

The authors apologize for this error and state that this does not change the scientific conclusions of the article in any way. The original article has been updated.

**Figure 2:**
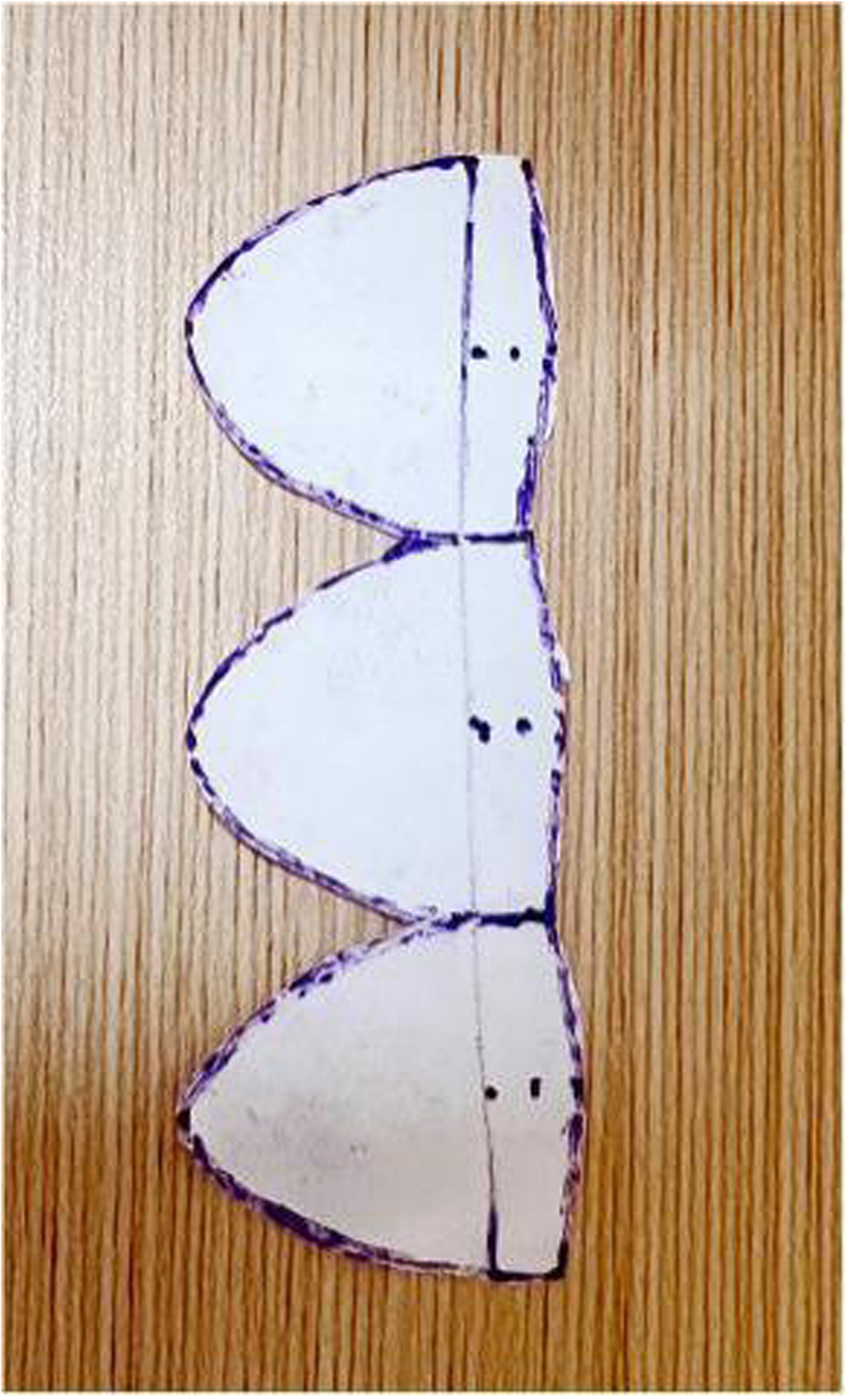
This figure shows the process of cutting out the valve leaflets as per the schematic diagram.

**Figure 3:**
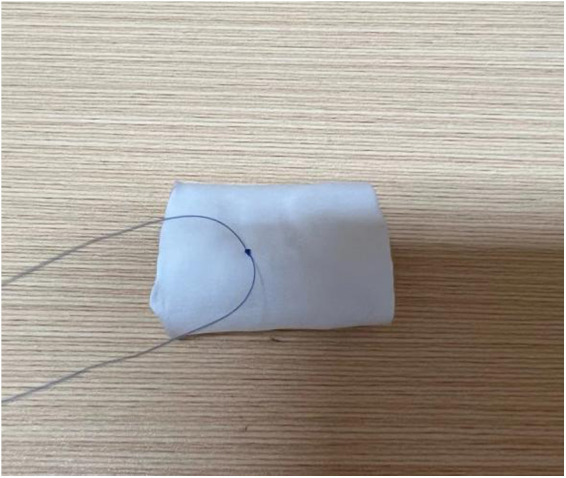
The image depicts the starting of the needlework from the junction where the two ends of the valve leaflets meet. A knot is tied at this starting point to secure the suture.

